# Correction: Badillo-Sanchez et al. Human Archaeological Dentin as Source of Polar and Less Polar Metabolites for Untargeted Metabolomic Research: The Case of *Yersinia pestis*. *Metabolites* 2023, *13*, 588

**DOI:** 10.3390/metabo15030187

**Published:** 2025-03-11

**Authors:** Diego Armando Badillo-Sanchez, Donald J. L. Jones, Meriam Guellil, Sarah A. Inskip, Christiana L. Scheib

**Affiliations:** 1 School of Archaeology and Ancient History, University of Leicester, Leicester LE1 7RH, UK; 2 Leicester Cancer Research Centre, RKCSB, University of Leicester, Leicester LE1 7RH, UK; 3 The Leicester van Geest MultiOmics Facility, University of Leicester, Leicester LE1 7RH, UK; 4 Institute of Genomics, University of Tartu, 51010 Tartu, Estonia; 5 Department of Evolutionary Anthropology, University of Vienna, 1030 Vienna, Austria; 6 McDonald Institute for Archaeological Research, University of Cambridge, Cambridge CB2 3ER, UK; 7 St. John’s College, University of Cambridge, Cambridge CB2 1TP, UK

## Addition of an Author

One contributor’s name was missing in the original version of the authorship of the paper [1]. The author is Meriam Guellil, who determined the metagenomic profile of the original samples, which was a key factor in choosing *Y. pestis* positive and negative samples for this study. As this paper contains some samples for which the *Y. pestis* positive status was not yet published elsewhere and thus contains new findings directly attributable to her work, she should be listed as the third author. She belongs to the original affiliation 4 and new affiliation 5 (Meriam Guellil ^4,5^):

4 Institute of Genomics, University of Tartu, 51010 Tartu, Estonia
5 Department of Evolutionary Anthropology, University of Vienna, 1030 Vienna, Austria

The updated authorship is as follows:

Diego Armando Badillo-Sanchez, Donald J. L. Jones, Meriam Guellil, Sarah A. Inskip and Christiana L. Scheib.

The corrected Author Contributions statement appears here:

Conceptualization: C.L.S.; data curation: D.A.B.-S.; formal analysis: D.A.B.-S. and M.G.; funding acquisition: C.L.S.; investigation: C.L.S., M.G. and D.A.B.-S.; methodology: D.A.B.-S. and C.L.S.; project administration: C.L.S. and S.A.I.; resources: S.A.I. and D.J.L.J.; supervision: S.A.I., C.L.S. and D.J.L.J.; visualization: D.A.B.-S.; writing—original draft: D.A.B.-S. and C.L.S.; writing—review and editing: D.A.B.-S., C.L.S., S.A.I. and D.J.L.J. All authors have read and agreed to the published version of the manuscript.

## Text Correction

There was an error in the original publication [1]. The Cambridgeshire County Council was incorrectly named the Cambridge County Council in the acknowledgements.

A correction has been made to the Acknowledgments section:

The authors also thank the support of the Cambridgeshire County Council.

The authors state that the scientific conclusions are unaffected. This correction was approved by the Academic Editor. The original publication has also been updated.
