# Supplementary material for: Human Archaeological Dentin as Source of Polar and Less Polar Metabolites for Untargeted Metabolomic Research: The Case of Yersinia pestis
Source: Metabolites. 2023 Apr 24;13(5):588. doi: 10.3390/metabo13050588 (PMC10223108; doi:10.3390/metabo13050588)
Supplement: Supplementary file 1 [file metabolites-13-00588-s001.zip › metabolites-2315186-supplementary file/metabolites-231586-supplementary file/Supplementary Data.docx]

# Supplementary Tables

**Table 1.** List of significatively up or down metabolites in the comparison of positive vs negative groups.

| *m/z* | *r.t. (min)* | *FC* | *log2(FC)* | *raw.pval* |
| --- | --- | --- | --- | --- |
| *721.4305* | 1.9289 | 3.5325 | 1.8207 | 0.00105 |
| *679.3331* | 5.209033 | 0.3791 | -1.3993 | 0.001588 |
| *155.0795* | 3.580383 | 2.4267 | 1.279 | 0.003352 |
| *661.3252* | 5.209033 | 0.42024 | -1.2507 | 0.00414 |
| *601.2897* | 5.07945 | 0.35929 | -1.4768 | 0.004786 |
| *239.4477* | 4.63985 | 2.4793 | 1.3099 | 0.005064 |
| *693.3967* | 1.979717 | 3.0453 | 1.6066 | 0.005528 |
| *171.0737* | 4.329883 | 2.5569 | 1.3544 | 0.005587 |
| *707.4153* | 1.9289 | 4.0103 | 2.0037 | 0.005621 |
| *198.0734* | 3.529567 | 2.1234 | 1.0864 | 0.006092 |
| *615.2972* | 5.07945 | 0.46153 | -1.1155 | 0.006927 |
| *572.2325* | 4.279067 | 4.2814 | 2.0981 | 0.011456 |
| *269.0585* | 4.589033 | 2.0535 | 1.0381 | 0.012175 |
| *558.4844* | 1.849933 | 2.0782 | 1.0553 | 0.01315 |
| *555.2521* | 5.07945 | 0.42981 | -1.2182 | 0.014687 |
| *609.3114* | 5.259833 | 2.0727 | 1.0515 | 0.016481 |
| *648.8184* | 5.209033 | 0.49895 | -1.003 | 0.019636 |
| *642.319* | 5.209033 | 0.38362 | -1.3823 | 0.021196 |
| *615.7923* | 5.209033 | 0.4536 | -1.1405 | 0.026734 |
| *335.1308* | 5.5698 | 2.1831 | 1.1264 | 0.034021 |
| *m/z* | r.t. (min) | FC | log2(FC) | raw.pval |
| *100.0739* | 3.270383 | 2.4227 | 1.2766 | 0.037046 |
| *362.6974* | 4.899067 | 0.47162 | -1.0843 | 0.037264 |
| *404.1893* | 4.1495 | 2.2433 | 1.1656 | 0.038217 |
| *645.3187* | 5.209033 | 0.47569 | -1.0719 | 0.040219 |
| *638.8186* | 5.259833 | 0.49975 | -1.0007 | 0.041368 |
| *284.0414* | 4.019917 | 4.4826 | 2.1643 | 0.049169 |

**Table 2.** Level 2 IDs from the HILIC column.

| Compound | Source / Description |
| --- | --- |
| Aeruginopeptin 228A | Produced by cyanobacteria (soil) |
| Yayoisaponin B | Plant derived (soil?) |
| Tragopogonsaponin N | Plant derived (salsify / soil?) |
| Lyciumin B | coffee, herbs and spices, and tea (soil?) |
| Tetrocarcin A | Produced by micromonospora bacteria (soil?) |
| Albanin H | Fruits (soil?) |
| Aclarubicin | Produced by soil bacteria (soil) |
| Galb1-3[Neu5Aca2,6]GalNAca-Thr | fluorogenic substrate used for testing the presence of glycoproteins in food samples (lab contaminant?) |
| Aminoparathion | anatidaes (Anatidae), chickens (Gallus gallus), and domestic pigs (Sus scrofa domestica) - soil? |
| 2-Octaprenyl-3-methyl-6-methoxy-1,4-benzoquinone | Intermediate in coenzyme Q biosynthesis produced by gut microbes |
| 3,5-Pyridinedicarboxylic acid, 2-(hydroxymethyl)-6-methyl-4-(3-nitrophenyl)-, 5-(2-hydroxyethyl) est | Unknown |
| 7-Piperazin-1-yl-thieno[2,3-c] Pyridine (hydrochloride) | synthetic intermediate useful for pharmaceutical synthesis (lab contaminant?) |
| Asp Val Arg Gly Asn | Unknown |
| Akeboside Ste | Human and up regulated in latent TB infections |
| Alliosterol 1-rhamnoside 16-galactoside | Lab contaminant / garlic/onion product (soil?) |

# Supplementary Figures


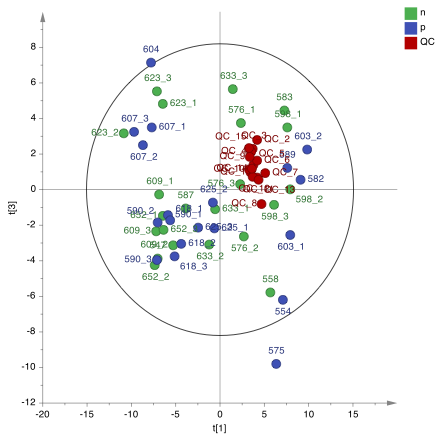

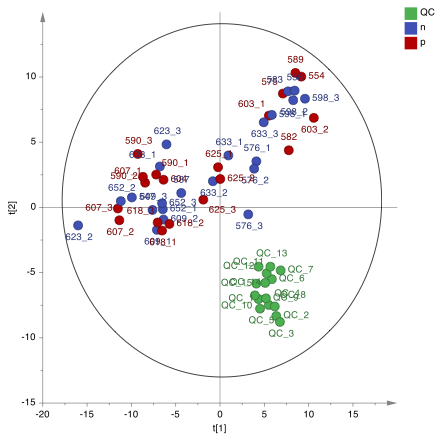


**Figure S1.** PCA scatter plot for the Raw data obtained after LC-HRMS measurements in the untargeted metabolomics assay for less polar/ apolar metabolites through reversed phase C18 column separation. Left panel scatter plot PC1 vs PC2. Right panel scatter plot PC1 vs PC3.


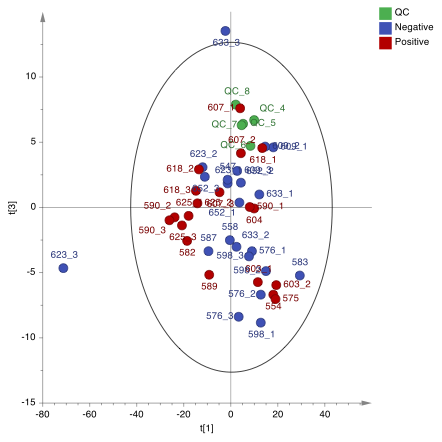

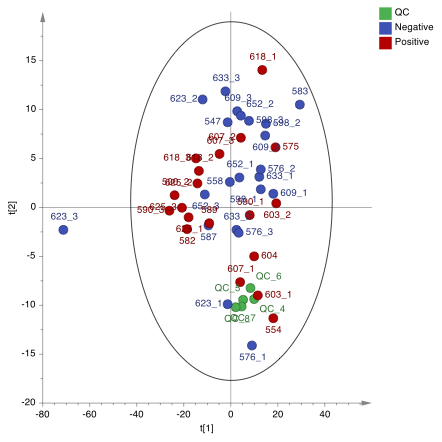


**Figure S2.** PCA scatter plot for the Raw data obtained after LC-HRMS measurements in the untargeted metabolomics assay for polar metabolites through HILIC column separation. Left panel scatter plot PC1 vs PC2. Right panel scatter plot PC1 vs PC3.


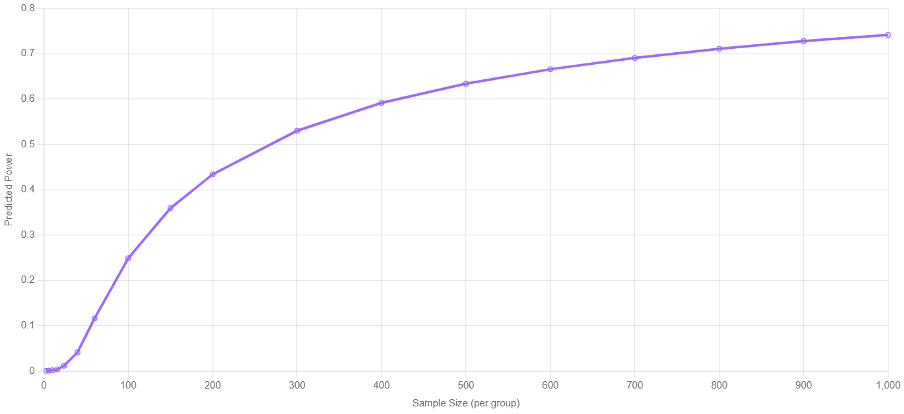


**Figure S3.** Sample size and power analysis plot for a polar metabolites untargeted metabolomics assay in archaeological dentine.
